# Unraveling the Relationship of Asthma and COVID-19

**DOI:** 10.3390/jpm11121374

**Published:** 2021-12-16

**Authors:** Agamemnon Bakakos, Petros Bakakos, Nikoletta Rovina

**Affiliations:** Department of Respiratory Medicine, National and Kapodistrian University of Athens, 11527 Athens, Greece; agabak@hotmail.com (A.B.); petros44@hotmail.com (P.B.)

**Keywords:** Asthma, COVID-19, SARS-CoV2, immunology, asthma treatment

## Abstract

Viral infections are one of the main causes of asthma exacerbations. During the COVID-19 era, concerns regarding the relationship of SARS-CoV2 with asthma have been raised. The concerns are both for COVID severity and asthma exacerbations. Many studies on COVID-19 epidemiology and comorbidities have assessed whether asthma represents a risk factor for SARS-CoV2 infection and/or more severe course of the disease. This review covers the current evidence on the prevalence of asthma in COVID-19 and its association with susceptibility to and severity of SARS-CoV2 infection. It will examine the possible role of underlying asthma severity in COVID-19 related outcomes as well as the molecular mechanisms involved in the co-existence of these entities. The possible role of asthma inflammatory phenotypes will also be evaluated. Finally, the impact of asthma comorbidities and the implications of asthma medication on COVID-19 will be addressed.

## 1. The Prevalence of Asthma in COVID-19

COVID-19 is a pandemic caused by coronavirus SARS-CoV2 that has so far affected 260 million people and caused almost 5.2 million deaths. Asthma is a common respiratory disease affecting almost 350 million people worldwide. It is characterized by shortness of breath, cough, chest tightness and/or wheeze that vary over time and in intensity.

There is great variation in the prevalence of asthma in the COVID-19 infected population among different countries (Table 1). This may be attributed to differences in the reporting rates, but mostly to the protective measures among respiratory disease patients, including asthmatics. The first studies coming from China demonstrated a low percentage of asthma when compared to asthma prevalence in the general population in the same areas [1,2,3]. The same was true for allergic comorbidities [4,5,6]. Later studies from Europe revealed contradictory data regarding asthma prevalence among hospitalized COVID-19 patients with some yielding low percentages (<2%), such as in Italy, while others showed higher prevalence (6%), such as in Spain [7,8]. In another study from Italy, a similarly low (<2%) prevalence of asthma was found among COVID-19 infected patients that required treatment in an intensive care unit [9]. More recent studies from the UK and the US revealed much higher rates of asthma among COVID-19 patients. An asthma prevalence of 14.4% was found in a US COVID-19 cohort, exceeding the prevalence of the disease in the general population [10]. In the same study, the prevalence of asthma among hospitalized patients with COVID-19 was 13.5% and no significant difference in the risk of hospitalization or mortality was detected in patients with or without asthma [10]. A similar percentage of 14% in asthma prevalence among COVID-19 patients was reported in the UK [11].

## 2. Asthma Exacerbations in COVID-19

Exacerbations are acute or subacute worsening of asthma symptoms that may be life-threatening and exert a significant effect on patients’ quality of life and public health [13].

Viral infections of the upper respiratory tract are one of the main causes of asthma exacerbations. Rhinoviruses are the most common cause of common cold [14] and have been more commonly related to asthma exacerbations [15]. Accordingly, the question arises as to whether SARS-CoV2 may be a cause of asthma exacerbations. Other coronaviruses are significantly less commonly reported as causes of asthma exacerbations compared to rhinovirus and RSV. It seems that SARS-CoV-2 rarely induces asthma exacerbations during hospitalization for COVID-19 [16,17].

## 3. Is Asthma Associated with More Severe COVID-19 Disease?

In one of the first studies in China—showing a very low asthma prevalence (0.9%)—no significant difference was observed in asthma prevalence between hospitalized patients with severe and non-severe COVID-19 disease [4]. A meta-analysis evaluating potential prognostic factors of mortality, which included 27 observational studies of patients with COVID-19, attempted to shed light on this topic. While particular comorbidities, such as coronary artery disease, congestive heart failure and COPD indeed increased mortality, asthma was not associated with an increased risk of severe disease and death from the novel virus [18]. Likewise, in a study from Italy, including 355 patients who eventually died of COVID-19, asthma was not reported among the comorbidities related to increased mortality [19]. No association was detected between asthma and mortality among COVID-19 patients in a study from the Mount Sinai Health System [12]. Conclusively, there are no robust data to support that asthmatics are more likely to become severely ill when infected with SARS-CoV2, and asthma is not associated with a higher risk of mortality (Table 2). It should be kept in mind that the definition of severe asthma is not unanimous among the various populations in different studies.

In accordance with the above, GINA suggested, in the interim guidance about COVID-19 and asthma, that people with asthma are not at increased risk of acquiring COVID-19 and that people with asthma are not at increased risk of COVID-19 related death [13].

Whether asthma severity plays a role in the risk of COVID-19 or the risk of severe COVID-19 and death is controversial. An index of asthma severity is the intensity of treatment required for asthma control. A retrospective study revealed that asthmatics treated with inhaled corticosteroids (ICS) plus long-acting beta agonists (LABA) presented a higher risk of hospitalization for COVID-19 compared to those treated only with ICS [10]. Another study, the OpenSAFELY Collaborative, demonstrated that among COVID-19 infected asthmatics, those who received high-dose ICS had a significantly higher risk of death compared to those who received combination therapy with long-acting beta-agonist (LABA) plus long-acting muscarinic agonists (LAMA) while no difference was observed in the risk of death between those who received low or medium dose ICS and those on SABA only [20]. Similarly, in patients with severe asthma on biologic therapy, no increased risk of COVID-19 infection or greater disease severity and mortality were found and no differences were detected among the different mAbs used for severe asthma [21,22]. Similarly, while a low asthma prevalence of 2.1% was found in an Italian cohort, including 2000 COVID-19 patients, GINA 4 and 5 step patients were more likely to have a worse outcome in this study (worse outcome defined as death, ICU admittance, and need for ventilator support) [23].

Compared to patients without asthma, asthmatics had significantly lower levels of ferritin, C-reactive protein, and lactate dehydrogenase. These laboratory markers have been used as markers of COVID-19 disease severity [10]. Such findings point towards a decreased inflammatory burden in asthmatic patients with COVID-19. However, whether this is due to the underlying asthma inflammation or due to asthma treatment is not yet clear. 

Various factors, such as the definition of asthma severity that is rarely assessed in everyday clinical practice accurately, the strictness of lockdown measures and the existence of other comorbidities may contribute to differences in the incidence of COVID-19 among severe asthmatics in different countries. 

In the SANI (Severe Asthma Network in Italy), including 1504 asthmatics, 1.3% suffered from COVID-19 and one third of them presented deterioration of asthma. In this study, severe asthmatics that were infected with SARS-CoV2 had a significantly higher prevalence of non-insulin dependent diabetes mellitus compared to those severe asthmatics that were not infected [24], indicating a role for comorbidities in SARS-CoV2 acquisition among patients with severe asthma.

## 4. Asthma Phenotypes and COVID-19

An important question arising is whether different asthma phenotypes, such as allergic and non-allergic asthma, may respond differently to the risk and severity of COVID-19 infection. A Korean cohort of 219,959 individuals of whom 7340 tested positive for SARS-CoV2 revealed that asthma, as well as allergic rhinitis, were associated with an increased likelihood of a positive SARS-CoV-2 test and worse clinical outcomes, including death, ICU admission and invasive ventilation. This study also reported that individuals with non- allergic asthma were more likely to test positive for SARS-CoV2 and had a greater risk for severe outcomes of COVID-19 than those with allergic (type 2) asthma [25]. Similarly, The UK Biobank study showed that asthmatics with non-allergic asthma had a higher risk of severe COVID-19 whereas patients with allergic asthma did not experience such an elevated risk for severe COVID-19 [26]. However, none of the above mentioned studies accounted for asthma severity, which may have confounded associations. Most recently, a large longitudinal study that included 434,348 asthma and 748,327 matched patients phenotyped asthma by medication, exacerbation history, and type-2 inflammation, using electronic medical records to identify asthma patient-level data, which were linked to Public Health England SARS-CoV-2 test data, hospital, and mortality data. This study demonstrated that patients with higher use of asthma maintenance treatment and more frequent exacerbations had significantly higher risk for severe COVID-19 outcomes, such as hospitalizations, ICU admission and death. Interestingly, no association was found between severe COVID-19 outcomes and variables representing probable type 2-inflammation, such as raised blood eosinophil count, atopy or history of allergic rhinitis [27]. 

## 5. Allergic Asthma as a Potential Protective Factor for COVID: Mechanisms Involved

The association of allergic asthma with viral infections (other than SARS-CoV2) has been extensively studied. A higher risk of viral infections in asthma has been associated with the lower interferon production and a synergism between allergen sensitization and viral infections in allergic asthma seems to play a role. Moreover, T2 high inflammation has been associated with impaired protection against viruses [14]. Type I interferons are antiviral cytokines that constitute the first line of defense against viruses. Most asthmatic patients are predisposed to produce lower levels of type I interferons upon respiratory viral infections, presenting a decreased defense against them. Allergic asthmatic children have increased FcεRI expression on plasmacytoid dendritic cells (pDCs) and after FcεRI cross-linking, they present significantly lower rhinovirus-induced IFN responses than allergic non-asthmatic and non-allergic non-asthmatic children [28,29]. pDCs from patients with asthma secreted significantly less IFN-alpha upon exposure to influenza A and secretion was inversely correlated with serum IgE levels. The crosslinking of IgE bound to FceRI diminished the influenza-induced IFN production and TLR-7 (Toll-like receptor 7) upregulation in pDCs, supporting a counter-regulation mechanism between FceRI and TLRs in pDCs and an impaired antiviral activity of pDCs upon IgE-cross-linking [30]. This deficient production of type I interferons in asthmatic patients, involving an inhibitory effect of the IgE crosslinking in pDCs has been further examined in a recent study which found that treatment with omalizumab (a mAb that blocks IgE) reduced asthma exacerbations and improved IFN-α responses to rhinovirus (RV). Within the omalizumab treated group, greater IFN-α increases were associated with fewer exacerbations [31]. Moreover, treatment with omalizumab decreased the duration of RV infection, reduced RV viral shedding and decreased susceptibility to RV infection [32]. Omalizumab also decreased the expression of FceR1a on pDCs, and such a decrease was linked with the restoration of RV- and influenza-induced interferon production leading to lowering asthma exacerbations [33].

SARS-CoV2 enters host cells–similarly to SARS-CoV1–via the angiotensin converting enzyme 2 (ACE2) receptor and this binding is facilitated by a transmembrane protease serine 2 (TMPRSS2). The affinity of SARS-CoV-2 for the ACE2 receptor is 10 times higher than that of SARS-CoV-1 [34]. A higher expression of ACE2 may increase the susceptibility to SARS-CoV2 infection [35]. A possible explanation for the observation that asthma and allergies are associated with reduced risk of infection and severe disease may be a reduced ACE2 gene expression in airway nasal and bronchial epithelial cells. In a study using nasal and bronchial allergen challenges, Jackson et al. [36] showed that asthma and allergic rhinitis as well as allergic sensitization were associated with reduced ACE2 gene expression. This reduction was more evident in cases of co-existence of asthma and high allergic sensitization whereas non-atopic asthma did not present reduced ACE2 expression [36]. In this way, allergic asthma, atopy and seasonal exposure to aeroallergens may protect from COVID-19. 

IL-13, a type-2 cytokine, has been shown to downregulate the expression of ACE2 on airway epithelial cells in asthma and allergic rhinitis [37]. Moreover, the same study revealed a significant negative correlation of IL-4, IL-5, and IL-13 (main type-2 cytokines) with ACE2 expression in the airway epithelial cells from participants with asthma [38]. On the other hand, IL-13 was associated with an increased expression of TMPRSS2 in nasal and airway epithelial cells in type-2 asthma and atopy [37]. The association of ACE2 and TMPRSS2 gene expression with type 2 cytokines was not dependent on the effect of inhaled and/or intranasal corticosteroids in this study. Conclusively, increased expression of TMPRSS2 may counteract any protective effect of reduced ACE2 expression. Non-type 2 asthma is characterized by TH1- type responses and TH17 cell activation. It should be noted that there might be an uncoupling between ACE2 protein and RNA expression, since in a mouse model ACE2 was shown to inhibit IL-17 and neutrophilic inflammation in the lungs [39]. Data on protein levels of ACE2 is necessary in order to confirm these observations. Eventually, in the Severe Asthma Research Program cohort (SARP), sputum gene expression of ACE2 was found to be lower than TMPRSS2, but expression levels of both genes were similar in asthma and healthy controls. Asthmatics that were male, African American and had a history of diabetes mellitus presented higher expression of ACE2 and TMPRSS2. On the other hand, ICS use was associated with a lower expression of ACE2 and TMPRSS2 [40].

Eosinophils play a pivotal role in asthma pathogenesis and blood eosinophilia is a marker for T2 inflammation [41]. Eosinophils contribute to innate antiviral immunity. They express Toll-like receptors (mainly TLR7) that recognize viral nucleic acids and are activated after stimulation from single- stranded RNA, thus providing host defense against respiratory syncytial virus (RSV) [42]. Since eosinopenia has been a biomarker for the severity of COVID-19 [43], eosinophil depletion induced by anti-IL5 and anti-IL5 receptor blocking monoclonal antibodies raises concern in patients and their treating physicians, despite the lack of evidence for increased risk of COVID-19 in asthmatics treated with mepolizumab, reslizumab or benralizumab [21]. 

An interesting theory for explaining reduced COVID-19 severity in allergic asthma is that of heterologous immunity. Heterologous immunity may alter the immune response to an infection with a pathogen as a consequence of a previous infection with another pathogen [44]. This may happen between closely related or completely unrelated antigens. In case of protein sequence homology between SARS-CoV2 and allergens, it is possible that cross-reactive T-cell epitopes are generated. In this way, patients with allergic asthma may carry in their T-cell repertoire T cells that are cross-reactive to SARS-CoV-2 and allergens. This may yield an advantage for patients with allergic asthma when infected with SARS-CoV-2. More studies are needed to support or refute this attractive theory.

In contrast to allergic asthma, non-type 2 asthma involves the activation of neutrophils, which drives the immune response toward a TH1/TH17 response [45]. Patients with COVID-19 present an immune response polarized toward a classic TH1 response, and the comorbidity of non-allergic asthma might aggravate such TH1 immune response. It has already been shown that there is a positive correlation between ACE2 gene expression and IL-17 (TH17) gene expression which may facilitate SARS-CoV2 infection [38]. Additionally, TH1/TH17 cytokines such as TNF, IL-12, and IL-17A can upregulate ACE2 expression [46]. Asthmatics with this endotype suffer more often from comorbidities, such as obesity, diabetes mellitus and hypertension [2], and it has already been mentioned that some of these comorbidities are associated with a higher expression of ACE2 and TMPRSS2 [40]. Eventually, patients with non-type 2 asthma present a low number of blood eosinophils and this may preclude the contribution of these cells to combat SARS-CoV-2 [47].

Although there are data to support an interplay of asthma phenotypes and underlying mechanisms with COVID-19, the majority of the so far published studies are retrospective and do not discriminate asthmatic patients into phenotypes. Better pheno/endotyping of patients with asthma and COVID-19 is required if we want to define more precisely the immunologic links between these two diseases.

## 6. Implications of Asthma Treatment on COVID-19 

There is increased interest on the adverse effects of asthma treatment on COVID-19 patients. It is well known that ICS are the mainstay of asthma treatment, while systemic corticosteroids are frequently used in severe refractory asthma and in the exacerbations of the disease. Currently, the suggestion for asthma and COPD COVID-19 patients receiving ICS is to carry on with their treatment [13]. 

However, the remaining question is whether premorbid use or chronic treatment with ICS is of benefit. Halpin et al. [48] did not reach a clear-cut conclusion on this question in their systematic review of patients with coronavirus acute respiratory infections (MERS, SARS and COVID-19). There is no definite association between ICS treatment and susceptibility to viral infections, on the contrary there are reports of a paradoxical protection against viral infections [49,50]. Specifically, ciclesonide has been shown to block SARS-CoV2 replication in vitro, inhibiting its cytopathogenicity [51,52]. This was indirectly supported in the study of Iwabuchi et al. [53] who showed an improvement of COVID-19 patients on oxygen therapy after treatment with inhaled ciclesonide. Antiviral and anti-inflammatory properties in vitro are reported for budesonide as well [54]. In an open-label, parallel group, phase 2 randomized control trial [55] in mild outpatient COVID-19 patients, those who received inhaled budesonide had a 91% relative risk reduction in COVID-19-related emergency department visits or hospitalization as compared to the standard of care. On the other hand, Schultze et al. [20] reported increased risk of mortality in asthma patients receiving high-dose ICS, acknowledging however, that this finding could be attributed to several confounders. 

Presently, there is cumulative evidence on the effects and safety of systemic corticosteroids on COVID-19 patients with or without concomitant asthma, after the positive results of the RECOVERY trial [56]. The National Institutes of Health (NIH), COVID-19 Treatment Guidelines and the World Health Organization (WHO) recommend dexamethasone treatment in hospitalized patients with respiratory failure requiring non-invasive/High flow oxygen device or invasive ventilation [57]. Several studies have associated systemic corticosteroid treatment with increased viral load, immune suppression, prolonged viral shedding, delayed viral clearance, and increased mortality [58,59,60,61]. However, there is scarce data on the effect of systemic corticosteroids on COVID-19 in asthma patients. Chhiba et al. [10] did not show any association between systemic corticosteroid use and COVID-19–related hospitalization. However, other studies demonstrated that chronic or recurrent systemic corticosteroid use relates with increased risk for severe COVID-19 and higher mortality. Using primary care electronic medical records, Williamson et al. [62] reported that COVID-19 patients with severe asthma having a recent use of systemic corticosteroids had increased mortality risk. More recently, Adir et al. [63] in a large nationwide study that included 80,602 asthma patients showed that systemic corticosteroids use was an independent risk factor for more severe COVID-19 and higher all cause 90-day mortality. However, they did not find a higher risk of viral infection in patients treated with systemic corticosteroids or biologics. These observations suggest that the more severe asthma is, the higher the risk of developing severe COVID-19 disease and the possibility to experience a worse outcome.

Biologic treatment (omalizumab, an anti-immunoglobulin E (IgE) antibody; mepolizumab and reslizumab, which block interleukin IL-5; benralizumab, which blocks the IL-5 receptor; and dupilumab, which blocks IL-4 receptor α, a receptor shared by IL-4 and IL-13) have all been approved for the treatment of severe asthma and have been shown to be effective in reducing symptoms and asthma-related exacerbations. 

In the face of the COVID-19 pandemic, two major concerns on the safety of the biologics are raised. Firstly, is there higher susceptibility of SARS-CoV2 infection in asthma patients that receive biologic therapy? Secondly, once infected, how would the biologic treatment affect the severity and outcome of COVID-19? Published data on the effects of biologic therapy on the risk of COVID-19 are controversial (Table 3). 

In a large study from Spain, including 545 severe asthmatics under biologic treatment, no increased risk of COVID-19 infection or greater disease severity and mortality was found and no differences were detected among the different mAbs used for severe asthma [21]. 

In a Belgian cohort, a low incidence of COVID-19 infection in severe asthma patients (2.1%) was noted and no severe disease or death occurred [22]. In accordance with the previous study, the use of biologics for severe allergic or severe eosinophilic asthma was not associated with a higher risk of COVID-19 [22]. In addition, Adir et al. [63] showed that severe asthma patients on biologic treatment were not at higher risk of SARS-CoV-2 infection, as compared with asthmatic patients that were not on biologic therapy. In contrast, two recent studies suggested a higher susceptibility of viral infection, especially in severe asthma patients on biologic treatment [63,65]. 

Biologic treatment may act protectively via several pathways. Omalizumab prevents IgE from binding to its receptor on plasmacytoid dendritic cells, leading to lower IFN-1 production by cross-linking of IgE, and to a decreased risk of duration, and viral shedding of human rhinovirus in children with allergy [28]. Furthermore, mepolizumab, reslizumab and benralizumab may also play a protective role in enhancing immune responses to viral infections, given that a higher ratio of IFN-γ/IL-5 mRNA is associated with lower viral shedding and faster clearance [66]. In addition, low eosinophil counts in peripheral blood may be related to the infection with the SARS-CoV-2 virus itself, and not necessarily be an indicator that treatments, which reduce eosinophil counts in patients with asthma. would be associated with in more severe COVID-19 disease. Interestingly, eosinopenia is not reported in patients with severe COVID-19 in recent studies from Italy and China [4,8,67]. 

Dupilumab, which blocks IL-4 receptor α, a receptor shared by IL-4 and IL-13, leads to decreased airway inflammation, allergic hyperresponsiveness and asthma exacerbations. Interleukin 4 is essential for antibody switching to IgE. Additionally, IL-13 is involved in airway hyperresponsiveness and remodeling, factors that are involved in susceptibility and clearance of lower airway viral infections [68]. Indeed, clinical trials with dupilumab do not report increased risk for viral respiratory infections [69,70]. In contrast, it is likely that dubilumab will rather lessen the risk of severe asthma exacerbations in patients with COVID-19, at least by reducing baseline airway inflammation, possibly through specific antiviral properties. 

Collectively, based on the current knowledge, it seems that biologic therapy for severe asthma rather acts protectively by limiting Th2 skewing, and preparing the immune system better poised to clear SARS-CoV-2. 

## 7. Conclusions

The prevalence of asthma in the COVID-19 infected population varies among different studies and countries. It seems that people with allergic asthma are not at increased risk of SARS-CoV2 infection and not at increased risk of COVID-19 related death. Additionally, underlying asthma severity is not a determinant of COVID-19 outcomes. In accordance, patients with severe asthma receiving biologic therapy presented neither increased risk of COVID-19 infection nor greater disease severity and mortality. Severe asthmatics should continue their regular maintenance treatment with ICS and even biologics in the COVID-19 era. Non-allergic asthma seems to present a greater risk for severe COVID-19 outcomes compared to allergic asthma. There are several potential mechanisms explaining why allergic asthma and atopy may be associated with reduced risk of infection and severe disease and on the other hand why non-allergic asthma may confer worse outcomes. Better endotyping of patients with asthma that are infected with SARS-CoV2 is of utmost importance in order to elucidate the immunologic links between these two diseases.

## Figures and Tables

**Table 1:** Studies reporting asthma prevalence in COVID-19.

Study	Country	Study Design, Population	COVID-19 Patients (*n*)	Asthma Prevalence
Li X., et al. [4]	China	Retrospective study of COVID-19 patients admitted to hospital	548	0.9%
Zhang J.J., et al. [5]	China	Retrospective study of hospitalized laboratory-confirmed COVID-19 patients	290	0.3%
Zhou F., et al. [6]	China	Retrospective, multicentre cohort study of hospitalized laboratory-confirmed COVID-19 patients	191	3% (with COPD)
Caminati M., et al. [7]	Italy (Brescia/Verona)	Retrospective study of hospitalized COVID-19 patients in two major Italian hospitals	1043/305	1.92%/1.96%
Grasselli G., et al. [9]	Italy	Retrospective case series of patients with laboratory-confirmed COVID-19 referred for ICU admission	1591 (available data in 1043 patients)	<2%
Borobia A.M., et al. [8]	Spain	Prospective study including patients consecutively hospitalized in a 1286-bed hospital of Madrid (Spain)	2226	5.2%
Chhiba K.D., et al. [10]	US	Retrospective analysis of medical records of PCR-confirmed COVID-19 patients	1526	14%
Docherty A.B., et al. [11]	UK	Prospective observational cohort study of hospital inpatients with COVID-19, using the report form developed by ISARIC and WHO	20,133	14.5%
Lieberman-Cribbin W., et al. [12]	US	Retrospective analysis of the MSHS COVID-19 registry	6250	4.4%

**Table 2:** Asthma and COVID-19 severity/mortality reports.

Study	Country	Number (%) of Total COVID-19 Patients Enrolled	Asthma Outcome
Li X., et al. [4]	China	269 (49.1) severe cases	1.1% severe cases
Zhou F., et al. [6]	China	54 (28.3) deaths in hospital	7% (deaths refer to chronic obstructive lung disease in general and not only asthma)
Borobia A.M., et al. [8]	Spain	460 (20.7) deaths in hospital	3.7% deaths in hospital
Shi C., et al. [18]	Metanalysis of 27 observational studies	2359 (infected)	RR of asthma mortality: 3.54
Onder G., et al. [19]	Italy	355 deaths in hospital	0
Lieberman-Cribbin W., et al. [12]	US	1128 (deaths)	4%

**Table 3:** COVID-19 and severe asthma on biologic therapy.

Study	Country	Asthma Patients on Biologic Therapy (*n*)	Asthma Patients on Biologic Therapy and COVID-19 (*n*-%)	Hospital Admissions * (*n*-%)	Deaths (*n*)
Rial M.J., et al. [19]	Spain	545	35–6.4%	8–22.9%	1
Heffler E., et al. [21]	Belgium	434	11–2.5%	4–36.3%	0
Adir Y., et al. [63]	Israel	8242	50–0.6%	-	HR of all-cause mortality within 90 days following PCR: 1.42 (0.70–2.88)
Izquierdo J.L., et al. [64]	Spain	865	20–2.3%	2–10%	1
Ferastraoaru D., et al. [65]	US	-	8–0.8%	6–1%	-

* Number and percentage of hospital admissions among those with severe asthma under biologic therapy that were infected with SARS-CoV2.
